# A Practical Treatise on Foreign Bodies in the Air Passages

**Published:** 1855-02

**Authors:** 


Art. III.—*A Practical Treatise on Foreign Bodies in the Air Passages.* By S. D. Gross, M. D., Professor of Surgery in the University of Louisville, etc., etc. With Illustrations. Philadelphia: Blanchard & Lea. 1854. 8vo., pp. 468.

---

When Dr. Gross first particularly directed his attention to the study of foreign bodies in the air passages, it was with the view of preparing a short monograph on the subject for this Journal. As he proceeded, however, with his researches, the subject grew in interest and extent until finally, in his hands, it has taken the form of a handsome octavo of nearly five hundred pages. We are informed in the preface that the materials of the volume have been derived, partly from personal observations, and partly from the experience of professional friends, but mainly from the various medical journals of the United States, Great Britain, and the Continent of Europe. The number of cases derived from the first two sources and which now, for the first time, meet the public eye, amounts to nearly fifty.

The labor involved in the task of this magnitude has been neither little or light. Dr. Gross found the whole subject in a state of chaos. He could turn to it in no tangible or connected form, but was obliged to seek his facts in the almost endless field of periodic literature.

Our author's first care was to collect the facts together; his second, to analyze and compare them with each other; and the third, to deduce from them such conclusions, general and particular, respecting the nature, symptomatology, pathology and treatment of foreign bodies in the air passages as the premises might fairly warrant. In so doing, he has prepared the first elaborate and complete monograph which has ever appeared, in any language, on foreign bodies in the air passages.

Chapter I. is devoted to the description of the nature, alterations, situation, entrance and expulsion of foreign bodies.

One cannot fail being surprised at the exceedingly diversified character of the bodies that may enter the air tubes. The catalogue collected by our author which comprises the most common and important of the bodies that have yet been found in these situations, contains the names of an almost infinite variety of substances in both the vegetable, animal and mineral kingdom. Arranging them into different classes, according to the nature of their composition, and observing the order adopted above, we have among the first, beans of almost every description; grains of corn, cockle burrs, nuts and nut-shells, sealing wax; tamarind, cherry, apricot and persimmon stones; charcoal, fiddle peg, &c., &c.

Of bodies derived from the animal kingdom our author enumerates bits of hard boiled egg, beef, veal, tendon, and bone; worms, leeches, locks of wool, teeth—natural and artificial—human and animal; flies, lobster claws, &c., &c.

The number of mineral substances that find their way into the air tubes is quite considerable; as buttons; pins; needles; tacks; nails; glass; bullets; marbles; gold, silver and copper coins, &c., &c.

Of all the substances that we have named and more than a hundred that we have not named, those which most commonly enter the air passages are grains of corn, beans, melon seeds, cherry stones, pebbles, and bits of meat, bone or gristle. "In the West, the first of these bodies are, perhaps, oftener found in the windpipe than any other." "In France, Germany, and England, the most common foreign bodies appear to be beans. Pieces of coin, pins, buttons, and similar articles, in consequence of the foolish habit, so common in almost every part of the civilized world, of holding such substances in the mouth, are often entrapped in the air-tubes."

Several foreign substances may enter the air-tubes either simultaneously or successively. A case occurred in the practice of our author in which a sprig of cedar was first inhaled, and about five weeks afterwards a piece of sewing silk, which were ejected at separate intervals. The most remarkable example perhaps, of the multiplicity of foreign bodies was reported to Dr. Gross by one of his former pupils. It happened in a child only eighteen months old. Larygotomy was performed. Six fragments of parched corn, one nearly the size of half a grain, were ejected immediately after the operation and the same number during the course of the same day and evening.

Foreign bodies of a vegetable or animal nature, acted upon by the heat and imbibing the moisture of the surface with which they are in contact, may become swollen and softened. Cartilage and tendon, apple, cabbage, turnip, carrot, and similar vegetable substances are exceptions to this rule. The same is true of hard boiled egg and roasted meats generally. The degree of this expansion and softening varies so much in different cases and in different circumstances as to admit of no precise statement. The particular situation of the foreign body has, it is thought, some influence upon the change of bulk and consistence effected during its sojourn in the windpipe. A substance impacted in one of the bronchial tubes would, in the opinion of our author, be likely to experience this change in a greater degree as well as more rapidly, than one lodged in the trachea or larynx.

Some practitioners, it seems, entertain most absurd notions respecting the softening power of the air-passages in this accident. A case is recorded where the question of operation was decided against on the ground that if the offending substance was a piece of gristle it might be softened and coughed up. Now while the softening process is sometimes carried so far as to break down the foreign substance, or to convert it into fragments, which are afterwards ejected separately, or *en masse*, we can by no means anticipate such a result save in very rare instances—at least, it can but be regarded as a most remote contingency.

If the foreign body be long retained, it often becomes encrusted with various kinds of matter. This is more likely to occur when it occupies the bronchial tubes than when it is situated in the larynx or trachea.

"The foreign body may be arrested in different portions of the windpipe, or it may remain loose, and move up and down the canal during the expulsion and introduction of the air. Occasionally it is stopped at the very entrance of the larynx; but more frequently, by far, it passes into the interior of this tube, and lodges in one of its ventricles.

How many persons have perished, perhaps in an instant, and in the midst of a hearty laugh, the recital of an amusing anecdote, or the utterance of a funny joke, from the interception at the glottis of a piece of meat, a crumb of bread, a morsel of cheese, or a bit of potato, without a suspicion, on the part of those around, of the real nature of the case! Many a coroner's inquest has been held upon the bodies of the victims of such accidents, and a verdict rendered that they died by the visitation of God, when the actual cause of death lay quietly and unobserved at the door of the windpipe of the deceased."

The foreign body may be arrested in the interior of the larynx, or it may descend into the trachea, or fall down into one of the bronchial tubes, more commonly the right. It is almost needless to remark that its disposition in any portion of the windpipe, will be influenced in a great degree, by its form, size and weight. Thus, for instance, a small and smooth body will, in general, be more likely to pass beyond the larynx than one that is large and rough, and a heavy substance than one that is light.

When the foreign body is arrested in the larynx, it may occupy one of the ventricles of Morgagni, or it may be engaged between the vocal chords. "Occasionally, though rarely, it is stopped in the inferior portion of the tube, or partly in the larynx and partly in the trachea."

The extraneous substance is not often arrested in the trachea, or if arrested does not long remain in it. Sharp and slender or very rough and angular bodies in consequence of their extremities becoming implanted in the walls of the trachea constitute occasional exceptions to this rule.

"The disposition of the foreign substance in the bronchial tubes varies according to a number of circumstances, of which the principal are the relative capacity of those tubes, or, rather, the situation and arrangement of the septum by which they are united to each other, and the configuration, volume, and weight of the intruding body."

It is a well ascertained fact that foreign bodies, upon arriving at the inferior extremity of the trachea, have a much greater tendency to pass into the right bronchial tube than the left. "The true cause, undoubtedly, is the peculiar position and arrangement of the *septum* at the root of the trachea, indicating the line of junction of the two bronchial tubes. This septum, spur, or ridge, to which from its situation, the term bronchial may be appropriately applied, is not, as will be seen hereafter, in the mesian line, but decidedly to the left of it, as may be shown by a perpendicular line extending from the centre of the rima of the glottis to the corresponding point of the inferior extremity of the windpipe, properly so called. Hence a body, especially one of considerable bulk, often having passed the larynx, will be very likely by striking this septum, as it will be apt to do in its descent, to be pushed over towards the right side, its entrance into the corresponding tube being still further favored by the greater diameter of this tube." The fact, of which the above is a most satisfactory explanation, is one of much importance in both a pathological and practical point of view. The question of the relative frequency of the situation of extraneous substances in different portions of the air-passages is, as our author remarks, one of great practical moment. An analysis of the cases contained in this report affords, concerning this point, the following results:—

"The number of cases of death, without operation and without expulsion of the offending body, is twenty-one. In these the substance was situated, in eleven, in the right bronchial tube; in four, in the larynx; in three, in the trachea; in one, partly in the trachea and partly in the larynx; in one, in the "lung;" and in one in the right thoracic cavity. In not a single instance did it occupy the left bronchial tube.

In thirty-four cases, subjected to operation, or general treatment, the extraneous substance was situated twice positively, and eleven times probably, in the right bronchial tube; four times certainly, and four times probably, in the left bronchial tube; seven times positively in the trachea, and fourteen times positively in the larynx. In two of the above cases a careful examination of the chest, during life, rendered it evident that the foreign substance, although found in the left bronchial tube after death, occupied the right tube of that name during the greater portion of the time which intervened between that event and the occurence of the accident."

When a foreign body is retained for any considerable period, it either becomes encysted, and is thus rendered comparatively harmless; or, as more generally happens, it sets up irritation in the surrounding parts, which in one case, perhaps, prepares the way for its own expulsion, while in another it may produce such ravages as to destroy the patient.

Foreign bodies do not, as might at first be supposed, always reach the windpipe by the route of the glottis. Occasionally they enter the tube from without, either by penetrating the skin and muscles of the neck, or by gaining ingress through an artificial opening, as in wounds of the throat. The amount of distress and danger consequent upon a foreign body entering the trachea from without is as great as when the extraneous substance is introduced by the natural channel.

"Foreign bodies sometimes lodge in the *pharynx*, and from thence pass into the larynx, either by their own efforts, or, more properly speaking, in consequence of the pressure exerted upon them by the neighboring parts, or in consequence of the means employed to extract them."

The glottis, as has been remarked, is the route by which foreign bodies usually enter the air-passages. They are generally expelled by the same course. The expulsion may occur spontaneously, it may be effected under the influence of various remedies, as emetics &c., &c , inversion of the body and bronchotomy. Whether by one means or the other there is no certainty in regard to the period of its occurrence. Bronchotomy itself does not always insure the immediate riddance of the foreign body.

Chapter II. contains an exceedingly satisfactory account of the *immediate effects of foreign bodies.* Chapter III. is devoted to the consideration of the *pathological effects of foreign bodies.*

The *Symptoms of foreign bodies* are detailed with great minuteness in chapter IV., and the *diagnosis of foreign bodies* forms the bulk of chapter V. *The spontaneous expulsion of*

*foreign bodies*, *the medical treatment*, *the surgical treatment*, *bronchotomy*, *tracheotomy*, *their dangers and difficulties* are treated of in chapters VI., VII., VIII., IX., and X.

These several subjects are illustrated by numerous cases, collected with great labor from many different sources. We cannot continue in detail our notice of these subjects without transcending our proper and original limits which we are unwilling to do since the gist of them all is contained in the following *general summary* which must bring this notice to a close.

From the numerous facts and cases adduced in the preceding pages, and from the reasoning founded upon them, the following conclusions may be fairly and legitimately deduced. These conclusions may be arranged under different heads, according to their respective relations, as diagnostic,, pathological, therapeutic and operative.

I. *Diagnostic Signs.*—Under this division of the subject may be briefly mentioned the nature and mode of entrance of foreign bodies, their liability to be arrested in different portions of the air-passages, and the symptoms commonly induced by their presence.

1. Any substance, whatever may be its form, provided it be not disproportionably large, may enter the larynx, and thence descend into the trachea and bronchial tubes.

2. The entrance of the foreign body is usually effected during a strong and sudden inspiration, while the epiglottis is off its guard, the glottis expanded, and the larynx quiescent.

3. The extraneous substance may be arrested in any portion of the air-passages, but not with equal frequency. Thus, in the larynx, it is, perhaps, most liable to be entrapped in the ventricles of Morgagni; and, when it descends into the bronchial tubes, it more frequently selects the right than the left. The trachea, on the contrary, rarely becomes its permanent receptacle; and the same is true in respect to the binary and tertiary divisions of the bronchial tubes.

4. The site of the foreign body is materially influenced, not generally, but frequently, by its size, weight, and configuration. Thus, a shot, ball, pea, bead, or pebble will be more likely to descend into the bronchial tubes than a rough, light, sharp, or angular substance.

5. The intruder may shift its place. Thus, it may pass from one bronchial tube into the other, or from these canals into the trachea, or from the trachea into the larynx, in a direction contrary to that of its entrance. On the other hand, it may be firmly impacted, either in its first situation, or in some secondary one.

6. The immediate and invariable effect of the entrance of a foreign body into the air-passages is a violent, spasmodic, and irresistible cough, with dyspnœa, and a sense of impending suffocation. The countenance is frequently livid, and the patient sometimes falls down in a state of insensibility.

7. The violence of the first symptoms continues from a few minutes to a quarter of an hour, half an hour, or even longer, when it is succeeded by a calm, variable in duration, and again followed by cough and difficulty of breathing, very much as in the first instance.

8. When the extraneous substance is arrested in the larynx, there will generally be more or less change in the voice, sometimes, indeed, total aphonia, hoarseness, and croupy cough, with diminished respiratory murmur in both lungs. The latter symptom will be most conspicuous when the body is so large as to impede materially the ingress of the air.

9. When the foreign body plays up and down the windpipe, as it often does when it is light and small, it always excites violent coughing and suffocative symptoms, very similar to those produced at the moment of its entrance. Under such circumstances, the patient can frequently feel the extraneous substance as it impinges against the trachea and the larynx. It is not so certain, however, as some have alleged, that the surgeon can hear and feel it by the application of the ear and fingers to the windpipe. Such an occurrence, at all events, must be very unfrequent.

10. A bulky body, relatively considered, may entirely fill the bronchial tube into which it may happen to fall, and thus give rise to complete collapse of the corresponding lung, as occurred in one of my own cases. A thin, flat body, as a coin, may produce the same effect, by acting as a sort of valve. In general, however, more or less air will pass by the side of it, thereby enabling the respiration to go on, although much more feebly than in the normal state. In both cases, the walls of the chest, on percussion, will emit a clear sound. The respiration in the opposite lung, after the first few days, will generally be puerile.

11. The site of the foreign body is occasionally, but not generally, indicated by a fixed pain, soreness, or sense of uneasiness in the larynx, trachea, or bronchial tubes.

12. The expectoration may be simply mucous, or it may be tinged with blood, or mixed with pus, especially in chronic cases, in which there is also occasionally hæmoptysis.

13. In attempting to establish the diagnosis of a foreign body in the air-passages, the practitioner must take into account, first the history of the case; secondly, he must carefully examine the condition of the respiratory organs, considering fully the rational and physical signs; and, thirdly, he must bear in mind the fact that the foreign body is liable to change its situation, thereby inducing corresponding alterations in the symptoms.

II. *Pathological Effects.*—These effects are primary and secondary; the former relating to what takes place immediately after the occurrence of the accident, and the latter to the organic alterations induced in the respiratory apparatus in consequence of the protracted retention of the foreign body.

1. The extraneous substance may destroy life at the moment of its introduction, or death may be induced at a variable period afterwards. In either case, the fatal effect may be produced by spasm of the larynx, or by mere mechanical occlusion.

2. The foreign body may be expelled immediately after its entrance, in a violent paroxysm of coughing, or it may be expelled subsequently either before or after it has induced serious structural lesion in the air-passages.

3. No patient is safe so long as the extraneous substance remains in the windpipe, whether in the laryngeal, tracheal, or bronchial portion, inasmuch as he may perish at any moment from suffocation, or, at a more or less remote period, from inflammation.

4. The danger from suffocation, when the patient escapes from the first effects of the accident, is generally greatest, all other things being equal, when the foreign body plays up and down the windpipe. If impacted, it may lead to the same result by becoming accidentally detached; but in such a case it will be more likely to destroy life through inflammatory action.

5. A foreign substance is occasionally comparatively harmless, as when, for example, it lies in one of the ventricles of the larynx; but, generally, it causes serious structural mischief.

6. The danger of severe and fatal inflammation is greater when the substance is lodged in the bronchial tubes than when it is arrested in the larynx or the trachea.

7. Violent and even destructive disease may be induced in the lungs, bronchial tubes, and pleura when the foreign body is situated in the larynx.

8. The most common structural lesion in cases in which the extraneous substance is retained for any length of time, are inflammation, abscess, and tubercles of the lungs, inflammation of the mucous membrane of the windpipe, and inflammation of the pleura, with effusion of serum and lymph, or sero-purulent matter. In rare instances there is marked alteration in the conformation of the chest, as happened in one of my own patients.

III. *Therapeutic and Operative Considerations.*—Under this head may be mentioned, aphoristically, the use of emetics, sternutatories, and other remedies, as expellents of foreign bodies; the importance of early recourse to bronchotomy, with the manner of performing the operation; and the various circumstances which should regulate the after-treatment.

1. Although foreign bodies have occasionally been ejected from the windpipe, under the influence of emetics, errhines, and other means, the number of such cases is too small to justify the practitioner, under any circumstances, in confiding in these different classes of remedies. Generally, indeed, their effect is to increase the respiratory suffering and the danger of the patient, by impelling the intruder against the larynx, where its presence always excites spasm and other unpleasant symptoms.

2. The remarks just made in reference to the use of emetics, sternutatories, and other means, are equally applicable to all spontaneous efforts at expulsion, whether they occur in the form of coughing, violent respiratory action, sneezing, vomiting, or dreaming; because, although these efforts are sometimes successful, yet they are more generally unavailing, if not positively hurtful, and even destructive to the patient.

3. Inversion and succession of the body, with or without beating of the chest, are generally hazardous proceedings, unless preceded by an opening in the windpipe; for the reason that the offending substance, if it be forced out of its lurking place into the larynx, or even against this portion of the tube, is inevitably followed by violent coughing and suffocative symptoms; thus greatly endangering the safety of the patient. The only case in which they ought to be practised, is where the foreign body is a shot, bullet, or similar substance.

4. Inasmuch, then, as no confidence is to be placed in the use of emetics, errhines, and other means, inversion and succussion of the body, and not even in nature's own efforts; and inasmuch, moreover, as no patient can be considered as being safe so long as the extraneous substance remains in the air-passages, it follows, as a necessary corollary, that bronchotomy affords the best chance of relief, and that, consequently, it should always be resorted to as early as possible, unless there is some special contraindication; as, for example, serious organic disease of the respiratory organs. The great danger in this accident is spasm of the glottis, which nearly always promptly disappears the moment the artificial opening has been effected.

5. In children, and in young or timid persons, the operation should always be preceded and accompanied by the administration of chloroform, which, while it perfectly calms the patient, greatly facilitates the extrusion of the foreign body, by rendering the respiratory organs tranquil and passive.

6. Laryngotomy is always comparatively easy of execution, and should, therefore, always be selected when there is a positive certainty that the intruder is lodged in the larynx. In all other cases, and also where the patient is very young and the neck very short, tracheotomy, although, in general, a very difficult procedure, should be preferred. Laryngo-tracheotomy is rarely necessary or proper, except in cases where the ordinary operations are found to be inadequate.

7. The windpipe, as a general rule, should never be opened before there is a cessation of hemorrhage, lest the blood, by falling into the tube, should embarrass the operator, if not seriously compromise the safety of the patient.

8. The opening, both in laryngotomy and in tracheotomy, but especially the latter, is generally too small to admit of the ready escape of the offending substance. To answer the purpose effectually, and this is one of the great objects of the operation, it should be at least one inch and a quarter in the adult, and not less than one inch in the child.

9. There is no necessity, in any case, for the removal of an elliptical portion of the trachea, inasmuch as the retraction of the edges of the wound by means of hooks will generally afford ample space for the ejection of the foreign body and the introduction of instruments. In laryngotomy, a crucial incision may sometimes be advantageously made into the crico-thyroid membrane.

10. Under no circumstances should bronchotomy be performed without a thorough exploration of the chest and œsophagus. It should be remembered that mere spasm of the glottis, caused by the lodgement of a foreign body in the fauces or gullet, or by derangement of the digestive, respiratory, and nervous functions, may induce a train of phenomena, closely resembling those occasioned by the presence of a foreign body in the air-tubes.

11. Bronchotomy is generally inadmissible when there is serious organic disease of the lungs, attended with marasmus and all the ordinary symptoms of pulmonary phthisis.

12. The foreign body, both in laryngotomy and tracheotomy, may escape either at the artificial opening, or by the glottis. In either case, it may be thrown to a considerable distance, perhaps the very moment the tube is pierced; or it may be intercepted by the edges of the wound; or it may, if it take the natural route, lodge in the mouth, or pass into the stomach.

13. Should the foreign substance not be ejected, or appear at the artificial orifice within a few minutes after the tube has been pierced, search should be made for it with the forceps, or hook, with a view to its extraction; but all such attempts should be made in the most gentle manner, nor should they be prolonged beyond a few seconds at a time; inasmuch as they almost invariably excite violent coughing and suffocative feelings. The use of chloroform will greatly facilitate this step of the procedure.

14. A much better plan than searching for the foreign substance, at least in the first instance, is to invert the patient's body, and to strike the chest with the hand, or with a pillow. This procedure should be tried in all cases of balls, shot, peas, beans, water-melon seeds, plum-stones, cherry-stones, button-moulds, and other similar articles. Inversion of the body, with previous opening of the tube, is a comparatively safe operation. Succussion and percussion are important auxiliaries in such a case.

15. When the extraneous body refuses to escape, or resists our efforts at removal, the edges of the tracheal wound should be kept apart by means of blunt hooks, in order to favor extrusion. The outer wound should be covered, in this case, with a piece of gauze, arranged in the form of a bag, to prevent the ingress of flies and dirt.

16. Riddance having been effected, the wound is closed with adhesive strips, aided, if necessary, by a few interrupted sutures, care being taken not to carry them through the substance of the trachea. Simple water-dressing is the best application, but even this may, in general, be dispensed with.

17. The after-treatment must be strictly antiphlogistic; the respiratory organs must be diligently watched; and the air of the patient's apartment must be maintained, throughout, at a uniform temperature, that is, at from 65° to 68° of Fahrenheit. It should be remembered that no patient is safe, or out of danger, after this accident, so long as there is inflammation of the respiratory organs, whether the intruder has been expelled or not.

18. Finally, it may be necessary to perform bronchotomy a second or even a third time. The same circumstances which induce us to perform it once may compel us to perform it again, at a more or less remote period, upon the same individual.

The work contains numerous illustrations which are rather well executed and add somewhat to its general interest. For sale in this city by Maxwell & Co.

